# A Treatise on Organic Nervous Diseases

**Published:** 1903-09

**Authors:** 


A Treatise on Organic Nervous Diseases. By M. Allen Starr, M. D., Ph. D., LL. D., Professor of Diseases of the Mind and Nervous System in the College of Physicians and Surgeons, New York. Lea Brothers & Co., New York and Philadelphia. 1903. (Price: Cloth, $6.00; leather, $7.00, net.)

The experience of 20 years upon which this work is based is so large and the observations of the author have been so accurate and painstaking that the deductions and conclusions reached by him are of great importance. The author's endeavor has been to give his own observation and experience in the presentation of each subject; hence, the book represents Dr. Starr and the statements found are those for which he is the authority. This makes the value of the book much greater than if the author had chosen that other and easier method, too much in vogue, of compilation and quotation.

The descriptions of diseases and the differential diagnoses are noteworthy features and present a certain definiteness of statement which aids greatly in weighing the value of a symptom or group of symptoms. One of the best examples of this is in the chapter on multiple neuritis, in which the differentiation of neuritis from anterior poliomyelitis, the neurotabes peripherica of Dejerine from locomotor ataxia, and neuritis from diffuse myelitis are models of clearness. In his directions for the use of hydrotherapeutics and electricity, the author has not made use of the general statements too frequently found in medical books. On the contrary, careful directions are given as to the manner of using the different baths. When advising the use of electricity, the size of electrodes, the strength of current and the duration of a treatment are carefully and minutely stated. This is a great improvement on the loose, vague statements that "currents of moderate strength should be used".

It is in the careful weighing of evidence for and against different methods of treatment that the physician will receive the greatest benefit. Especially is this true of his estimate of the various surgical procedures for the relief of nervous diseases and also for the relief of the different deformities resulting from nervous disease. This work is so preëminently the result of the clinician that it must for many years be a guide in the application of surgical procedures for the alleviation of diseases hitherto incurable.

Of Horseley's advice, that in cases of inaccessible brain tumor a large opening be made in the skull and that a drainage-tube be passed within the dura, or that the ventricle be drained to relieve, if possible, the intracranial pressure, Starr says:

"I have seen this done, but it seems to me of problematical value, as it merely prolongs the misery in a hopeless condition".

Of Lannelongue's operation of craniectomy, for the relief of microcephalic or epileptic children, he says:

"In my own collection of cases, 50 in number, the result cannot be said to be encouraging,—the dangers are many. The prospect of relief is small; cure cannot be promised. Improvement in a very small proportion of the cases only, is the best result which can be promised".

Starr is positive in his endorsement of exsection of the Gasserian ganglion for the relief of the chronic cases of tic douloureux. Of the surgical treatment of sciatica by nerve stretching, he is less hopeful. He has seen one cure and several failures. He believes that no positive promise of relief should be given. He takes a strong position in favor of early operation in spinal cord tumors, stating that a prompt surgical interference cannot be too strongly urged. The chapter on the diagnosis of brain disease is one of the most important.

The entire subject of the principles of cerebral localisation is reviewed and brought up to date. The book is profusely illustrated. The engravings and plate have been selected with great care; many of them are original and many are taken from European authors,—notably from Dejerine, Flatau, and Schmaus.

Too much praise cannot be given the publishers for the beauty of their work. They have succeeded in presenting in most attractive form the best book on organic nervous diseases. J. W. P.

